# Generics versus brand-named drugs for glaucoma: the debate continues

**Published:** 2020

**Authors:** Shibal Bhartiya, Deepika Dhingra

**Affiliations:** * Glaucoma Services, Fortis Memorial Research Institute, Gurugram, Haryana, India; ** Shah Satnam Ji Speciality Hospitals, Sirsa, Haryana, India

**Keywords:** generic, branded generic, brand name drug, glaucoma, drugs

## Abstract

There are three options for a given class of drugs, including brand name drugs, generic and branded generic drugs. Brand name drugs are costlier as compared to generic and branded generic drugs because they are innovator molecules developed by a company after many years of research and come into the market with a patent, whereas branded generic drugs are produced by a different company once the patent of innovator company expires. Given that glaucoma is a chronic, largely asymptomatic disease, the choice of drugs is extremely important: the duration of medication is often lifelong, and the cost of drugs, side effects and efficacy affect compliance and adherence to therapy.

This review is a brief overview of the available brand name and branded generic drugs for the management of glaucoma, in terms of efficacy and side effect profiles. It also aimed to guide rational and pragmatic drug choices in different clinical scenarios.

## Introduction

Given that glaucoma is largely an asymptomatic and chronic disease, the efficacy of topical medicines may be determined by several intrinsic and extrinsic factors including drug efficacy, variability in active ingredient concentration, patient adherence to, and side effects of prescribed therapeutic regimens, as well as the cost of therapy. 

As glaucoma therapy becomes increasingly costlier, the driving and restraining forces for therapeutic decisions are often economic. That is why, in choosing a glaucoma treatment regimen, generic formulations have to be considered as an essential part of the treatment paradigm. 

However, regulatory authorities, including the US FDA do not require a strict demonstration of human bioequivalence and/ or therapeutic equivalence studies for innovator or brand name drugs and generic formulations. The former is presumed to be a direct consequence of similar active and inactive ingredient profiles. Thus, generic formulations may not be consistently comparable in terms of drug composition, efficacy, and clinical equivalence. 

In addition, their use must be considered judiciously, especially since economic considerations often prompt their popularity. The implications of shifting from branded drugs to generic formulations require further studies, and these patients must be monitored more carefully, keeping in mind the possibility of decreased efficacy, and a higher incidence of side effects.

This review was an attempt to aid a pragmatic approach to this therapeutic decision making in current glaucoma practice. 

## Available therapeutic options 

There are 3 types of medicines for a given class of the drug: brand name, branded generic and generic formulations.

A brand name drug is an innovator molecule prepared by a pharmaceutical company through research and goes through rigorous clinical trials and regulatory approval before marketing. These drugs are costly as the companies have to cover the cost spent on drug studies, research, and marketing during the limited period of patent. 

Once the patent of the innovator expires, other pharmaceutical companies can manufacture a similar molecule and sell the drug with a different trade name. These are known as branded generics. They are less costly than brand name drugs as they did not have to spend for drug development and research. The cost further decreases in view of growing competition amongst various companies to make similar molecule at cheaper rates. 

Similarly, many pharmacies produce the same molecule, and the drug is sold with its chemical name and is known as generic medicines. Generic drugs have to be prepared with the same dosage, route of administration, same active ingredients as of innovator molecule, and need FDA approval before marketing. Generic drugs need to be bio-equivalent, and also demonstrate therapeutic equivalence [**1**,**2**]. Bioequivalence is demonstrated by showing equal absorption and drug levels in the blood, while therapeutic equivalence implies similar efficacy as well as safety [**3**].

## Ophthalmic drugs different from systemic drug: 

Since testing for bioequivalence and therapeutic equivalence is not feasible with ophthalmic drugs, generic formulations only have to demonstrate pharmaceutical equivalence. These drugs get approval if active and inactive ingredients are within ±5% of the level found in innovator formulation [**4**-**6**]. 

## Antiglaucoma medicines (AGM) different from other ophthalmic drugs:

Since glaucoma is a largely asymptomatic, chronic disease, AGMs are required for long-term use. Therefore, the cost, efficacy, and side effects of AGMs are of great concern for the patients as well as the treating doctors. Thus, in the context of AGMs, it is of great importance to know whether generic or branded drugs can be used interchangeably.

## GENERIC DRUGS VERSUS BRANDED DRUGS

**A. Therapeutic Equivalence and Safety**

Generic drugs are different from branded drugs in terms of inactive ingredients (buffer, excipients, preservative) and can have different bottle design, cap colour, labelling, etc. Thus, the side effects may be different. The cap colour, squeezability of the bottle, labelling instructions, are very important for many patients. Elderly patients who identify the bottle by colour may find it difficult to use them [**7**]. Similarly, the squeezability of the bottle can affect drug compliance. There are various studies on the comparison of generic versus brand name drugs, which have reported different pH, osmolarity, concentration of the drug, drop size, higher particulate matter in generic drugs, and decrease in their efficacy when stored at higher temperature of 25-50°C [**8**-**10**]. Regarding the efficacy of generic drugs, there are various reports in the literature. Some of the studies report equal efficacy; however, other studies report them to be marginally inferior.

Most of the literature available is about prostaglandin (PG) analogues, which are the first line drugs for glaucoma therapy because of their longer action and better intraocular pressure (IOP) reduction. Also, predictably, the use of PG analogues has gradually increased, while that of beta blockers has gradually decreased over the years [**11**]. The formulation of prostaglandins and carbonic anhydrase inhibitors are more difficult to prepare compared to beta blockers and miotics because of their lipophilicity.

## Prostaglandin Analogues: 

**Latanoprost:**

In a randomized double-masked multicentric study, generic latanoprost was found equivalent to Xalatan (Pfizer, New York, USA) in terms of IOP control with similar side effects in both the groups at the end of 12 weeks treatment [**12**]. 

In their crossover study (after 12 weeks period) to determine the safety and efficacy of branded generic Latanoprost (Latoprost; Sun Pharmaceuticals Industries Ltd., Mumbai, India) versus Xalatan, Narayanswamy et al. [**13**] have reported better IOP reduction with Xalatan (38.66%) than the non-innovator latanoprost (25.42%). The authors also reported that after shifting from Xalatan to the branded generic, there is a significant rise in IOP (8.86%) and vice versa, a significant fall in IOP (4.3%) when shifted from the branded generic drug to Xalatan. The side effects profile was similar in both groups. 

In another study, Kim et al. [**14**] reported a better efficacy with generic latanoprost in comparison with other branded PG analogues. This was a retrospective study in which authors reported a better efficacy of generic latanoprost in terms of less requirement of second drug and glaucoma procedures. 

A pilot 4-week crossover study of Xalatan versus generic Latanoprost by Egan et al. [**15**] on 35 patients of primary open angle glaucoma reported an equal efficacy with both the drugs, but the treatment with Xalatan had more patients with IOP < 14 mmHg compared to generic Latanoprost. Thus, in cases of moderate-advanced glaucoma, one has to be careful while shifting from brand drug to generic.

Golan et al. [**16**] compared the efficacy and safety of generic Glutan (Latanoprost, Unipharm, Israel) and Xalatan with crossover of drugs after 4 weeks of treatment, and a 3-week washout period. The authors reported better IOP reduction and lesser side effects with Xalatan, but the difference was statistically non-significant. Glutan patients had a higher incidence of irritation, grittiness, light sensitivity and eye pain.

Painter et al. [**17**] looked at a questionnaire-based patient experience when they were shifted from Xalatan to generic drug. In their study, the patients found a Xalatan bottle easier to open with easier instillation of drops and more comfortable and more patients preferred Xalatan compared to the generic formulation (75% versus 22%). In 20% of the patients, generic bottles did not last for 1 month.

Diagourtas et al. [**18**] prospectively compared the efficacy and safety of Xalatan with two generic drugs (Lataz, Rafarm Pharmaceuticals and Xaloprost, Cooper Pharmaceuticals, Greece) over 16 weeks in a newly diagnosed open-angle glaucoma patient. The authors found equal efficacy of all three drugs (30-32% IOP reduction), but better safety of Xalatan in terms of tear film breakup time (mean decrease of 0.47 seconds versus 0.7 seconds and 1 second with Xalatan, Lataz and Xaloprost respectively) and Ocular Surface Disease Index (OSDI) questionnaire with better tolerance with Xalatan.

**Travoprost:**

Ta Kim et al. [**19**] evaluated the efficacy and safety of generic versus branded Travoprost in a crossover trial (after 3 weeks), which has reported equal efficacy and side effects with generic Travoprost (Sandoz Canada Inc., Boucherville, Canada) and Travatan Z (Alcon Canada Inc, Mississauga, Ont.).

## Beta- Blockers 

**Timolol:**

Schenker et al. [**20**] compared branded Timoptic XE (Merck & Co., Inc., Pennsylvania, USA) with generic Timolol gel-forming solution (Alcon Pharmaceuticals, Ltd., Fort Worth, Texas, USA) and found similar IOP reduction with both the drugs and similar side effect profiles.

In a multicenter study, branded Isatolol (ISTA pharmaceuticals, Inc., Irvine, CA), which is timolol maleate with potassium sorbate preservative administered once daily, was compared with generic timolol maleate twice daily. The study results reported similar efficacy of both drugs. However, there were more cases of stinging with Isatolol (41.6% versus 22.9%, p- 0.001), which was mild in most of the cases [**21**]. 

## Fixed Drug Combinations (FDCs): 

Kim et al. [**22**] reported that a shift from brand drug Cosopt (dorzolamide + timolol, Merck and Co, Inc., USA) to generic drug Batidor (Bausch & Lomb, Inc., Canada) had equal IOP levels before and after switching the medicines without any discomfort.

Another study by Ali Aljasim et al. [**23**] on the comparison of brand drug Cosopt with generic drug Xolamol (Jamjhoom pharma, Saudi Arabia) in a crossover trial, reported equal IOP levels, but more cases of increased conjunctival congestion and punctate keratopathy with the generic drug.

**B. Cost:** Cost is the most important difference between generic versus branded drugs. Shifting from a branded/ branded generic drug to a generic drug usually saves considerable costs. This is especially relevant both for the out of pocket expense for the individual patient, and also from a public health perspective. 

However, patients often doubt the efficacy of the generic drug and many times they get different bottles or sometimes drugs are out of stock in pharmacy [**24**]. Therefore, the compliance of many patients decreases when shifting to the generic drug. In direct contrast to this, compliance and adherence may paradoxically increase, especially in poor patients who cannot afford costly medications [**25**,**26**]. 

There is a need to improve the usage of generic drugs to decrease health- related expenditure. Generic drugs are not inferior drugs and most of the studies have reported a nearly equivalent efficacy. As generic drugs can have different cap colours, it is important to ask patients to bring their medicine bottles with them at every visit so as to ensure proper medicine usage [**27**]. Several insurance companies only provide reimbursement on generic drugs only if they are available, thus it is important to be aware of these drugs. 

**C. Side effects:** As the data for generic drug efficacy is different across countries, the same holds true for the side effects. Various studies have reported similar adverse effects, whereas some of the studies have reported marginally higher incidence of side effects, including ocular irritation, conjunctival hyperemia, etc., with generic medicines [**13**,**22**]. There are isolated reports of corneal epithelial disorder with punctate keratopathy developing after shifting from a brand drug to a generic drug and resolution after shifting back to the branded latanoprost [**28**].

## How to choose a drug in clinical practice? 

As in the case of any therapeutic decision in glaucoma practice, the choice of branded or generic drugs must be customized. In poor patients and in developing nations, as also for public health initiatives, where cost is a big barrier in management, generic drugs must be the first choice. For patients for whom the cost is not a factor, and for whom efficacy and safety are of greater concern, branded drugs may be the first choice. In a stable glaucoma patient as well, a trial of generic drugs may be given in order to bring down the eventual cost of therapy. This may be continued if a therapeutic equivalence can be established for the individual patient in terms of both efficacy and tolerance. Conversely, a patient uncontrolled on generic medication, or having side effects from the same, may be offered a trial of the brand name formulation. In case the therapeutic profile is considerably different, the patient may prefer to continue with the brand name drug. 

In all circumstances, an open and frank physician-patient discussion about disease type, need of regular follow-up, and cost of life long therapy may influence compliance and adherence and is essential for any therapeutic decision making. Thus, a tailored approach and shifting of drugs may be considered to provide safe and cost-effective treatment. 

In addition, given that the drug efficacy and side effects of generic formulations may vary considerably from the brand name drug, there is a need for more stringent quality control from the regulatory authorities. This will positively affect their usage and acceptability by glaucoma practitioners and patients alike [**29**].

## Conclusion

The choice of drug type (brand name drug, generic or branded generic drug) for the management of glaucoma is guided by various factors including the stage of glaucoma, socio-economic status, patient expectations, efficacy and side effect profile of the drug. Many of the generic and branded generic drugs have been shown to have similar efficacy as of brand name drugs with a similar or marginally higher incidence of side effects. These drugs may be judiciously used in early-moderate glaucoma, stable disease course and with caution in advanced glaucoma, however, in a patient with intolerable side effects and inadequate IOP lowering and affordable patient, brand name drugs may be the preferred option. As it is true for all decisions in glaucoma practice, it is important to discuss all available choices with the patient and ensure that they are a part of the decision-making process.

**Sources of funding**

None.

**Disclosures**

Authors have nothing to disclose.

**Conflict of interest**

None.
